# The Genomic Intersection of Oligodendrocyte Dynamics in Schizophrenia and Aging Unravels Novel Pathological Mechanisms and Therapeutic Potentials

**DOI:** 10.3390/ijms25084452

**Published:** 2024-04-18

**Authors:** Andrea D. Rivera, John R. Normanton, Arthur M. Butt, Kasum Azim

**Affiliations:** 1 Department of Neuroscience, Institute of Human Anatomy, University of Padova, Via A. Gabelli 65, 35127 Padua, Italy; riveraandrea83@yahoo.it; 2 GliaGenesis Limited, Orchard Lea, Horns Lane, Oxfordshire, Witney OX29 8NH, UK; john.normanton@gliagenesis.com (J.R.N.); kasumazim@gmail.com (K.A.); 3 School of Pharmacy and Biomedical Science, University of Portsmouth, Hampshire PO1 2UP, UK; 4 Independent Data Lab UG, Frauenmantelanger 31, 80937 Munich, Germany

**Keywords:** oligodendrocytes, schizophrenia, white matter, aging, oligodendrocyte precursor cells/OPCs, multiple sclerosis

## Abstract

Schizophrenia is a significant worldwide health concern, affecting over 20 million individuals and contributing to a potential reduction in life expectancy by up to 14.5 years. Despite its profound impact, the precise pathological mechanisms underlying schizophrenia continue to remain enigmatic, with previous research yielding diverse and occasionally conflicting findings. Nonetheless, one consistently observed phenomenon in brain imaging studies of schizophrenia patients is the disruption of white matter, the bundles of myelinated axons that provide connectivity and rapid signalling between brain regions. Myelin is produced by specialised glial cells known as oligodendrocytes, which have been shown to be disrupted in post-mortem analyses of schizophrenia patients. Oligodendrocytes are generated throughout life by a major population of oligodendrocyte progenitor cells (OPC), which are essential for white matter health and plasticity. Notably, a decline in a specific subpopulation of OPC has been identified as a principal factor in oligodendrocyte disruption and white matter loss in the aging brain, suggesting this may also be a factor in schizophrenia. In this review, we analysed genomic databases to pinpoint intersections between aging and schizophrenia and identify shared mechanisms of white matter disruption and cognitive dysfunction.

## 1. Introduction

The World Health Organization (WHO) cites schizophrenia as affecting approximately 1 in 300 (24 million) people worldwide (0.33%) (https://www.who.int/news-room/fact-sheets/detail/schizophrenia# (accessed on 10 January 2022)), and the National Institute of Mental Health (NIMH) cites international prevalence amongst non-institutionalized persons as high as 0.75% (see also: https://www.nimh.nih.gov/health/statistics/schizophrenia (accessed on 10 January 2022)). Schizophrenia is one of the 15 leading causes of disability, and its prevalence is close to that reported for all forms of dementia, including Alzheimer’s disease. Schizophrenia manifests as a progressive decline in cognitive and functional abilities, culminating in aberrant behaviour, debilitating symptoms, and premature mortality. Key physical symptoms characteristic of schizophrenia include, but are not limited to (1) paranoid schizophrenia, featuring delusions, anxiety, anger, and hallucinations; (2) catatonic schizophrenia, characterised by agitation, negativism, limited communicative responses, catalepsy, and stupor; (3) disorganised schizophrenia, typified by flat affect, speech disturbances, disorganised thinking, inappropriate emotions, repetitive behaviours, and difficulties with daily routines; and (4) undifferentiated schizophrenia, which may encompass one or several of the aforementioned symptoms. Both males and females are equally susceptible to this disorder [1,2].

Mounting evidence suggests that changes in glial cells may be more prominent than those of neurons in schizophrenia and have been highlighted in key recent reviews [3,4,5]. In this mini-review, we focus on oligodendrocytes (OLs) in the underlying pathogenesis of schizophrenia. OLs myelinate axons in the central nervous system and are essential for ensuring rapid transmission of electrical signals, as well as supporting axonal metabolism [6,7]. The genesis of OLs and myelin, primarily from oligodendrocyte progenitor cells (OPCs), is a dynamic process that has been argued to persist throughout life, adjusting to meet the demands for myelin maintenance and repair [8,9]. This ongoing generation of OLs from OPCs, which are ubiquitously present within the adult brain and spinal cord, is essential for sustaining neural circuitry’s efficiency and plasticity [10,11,12]. These processes are sensitive to a myriad of internal and external stimuli, thereby facilitating adaptive responses to neuronal activity and compensating for myelin loss due to injury or disease [13,14,15]. OLs originate from OPCs through a regulated differentiation pathway, ensuring a balance between the production of new OLs and the preservation of the OPC pool for future needs [16,17,18]. However, for reasons unknown across the different myelin pathologies, the capacity for OPC differentiation drastically declines during aging as we and others have described [19,20,21]. In this mini-review, we underline the potential commonalities of aging and schizophrenia by highlighting shared cellular programs that may in turn inform the development of new avenues of targeted treatment strategies for this complex and debilitating disorder.

### 1.1. The Impact of Aging on OLs

As highlighted earlier, OL progenitor cells (OPCs) are vital for brain health, playing a crucial role in the maintenance and regeneration of myelin throughout life. Aging affects the capability of OPCs to evolve into myelinating OLs, a phenomenon well-documented across numerous studies. This review focuses on mechanisms observed in both aging and schizophrenia, given the extensive discussion of OPCs in the context of aging elsewhere (refer to citations in the previous section). Research, including proteomic comparisons of OPCs from neonatal, young, and aged rodents and various independent transcriptomic studies, reveals consistent alterations in myelin, cholesterol biosynthesis, transcription regulation, cell cycle, oxidative phosphorylation, and actin cytoskeletal organisation [22,23,24,25]. This evidence, supported by a wide range of literature, highlights the significant impact of aging on OPC functionality [18,19,20,26].

Furthermore, the effects of aging on myelin plasticity extend beyond OL differentiation. The accumulation of myelin debris in the aging brain may impede the remodelling of myelin sheath lengths and axonal plasticity, affecting the stability of the nodes of Ranvier. Notably, myelin unpacking can occur independently of OL loss, leading to impaired action potential transmission. Age-related disruptions in the metabolic support provided by OLs to axons, potentially due to variations in GLUT-1/MCT-1 density within myelin sheaths [27], underscore the importance of thoroughly investigating how aging influences myelin plasticity and the applicability of these findings to different neuropathological conditions. Myelin degradation, due to its critical and widespread functions, contributes to cognitive decline in the absence of clinical age-related pathologies, such as dementia. This leaves axons vulnerable to damage, akin to demyelinating conditions like multiple sclerosis [28]. Longitudinal diffusion tensor imaging (DTI) studies demonstrate that age-related myelin degradation significantly impacts cognitive function by causing cortical disconnection and reducing processing speeds [29,30], distinct from axonal degeneration [31]. Crucially, processing speed influences working memory efficiency [32], and recent studies have shown that toxin-induced demyelination in the hippocampus impairs learning and memory in rodents by decreasing neurogenesis [33,34]. Thus, white matter loss and degeneration can drive age-related cognitive decline through several distinct mechanisms.

While this review primarily addresses the normal aging process of the brain, aside from schizophrenia-specific discussions, it is critical to recognise that aging is the foremost risk factor for neurodegenerative diseases like Alzheimer’s. The growing evidence of white matter disruption in such diseases calls for a closer examination of white matter pathologies alongside traditional studies of grey matter [35,36]. The interplay between neuronal loss and myelin damage in Alzheimer’s and other age-related degenerative conditions presents a complex domain that is yet to be fully explored [37,38,39], highlighting the need for further comprehensive studies to elucidate these mechanisms.

### 1.2. Further Evidence of the Impact of Schizophrenia and Aging on White Matter from Imaging Studies

Cross-sectional longitudinal studies have identified significant grey and white matter alterations in schizophrenia [40,41,42]. Brain aging, as determined from imaging analyses, was found to be accelerated by at least 5.5 years compared to the chronological age of an unaffected brain [43,44]. Furthermore, life expectancy is reduced by 14.5 years in schizophrenia, correlating with the severity of disease progression and brain atrophy [43]. Intriguingly, white matter tracts associated with cognition are the most affected in schizophrenia, leading to the provocative hypothesis of “accelerated aging” in schizophrenia [40,45]. The hypothesis posits that the same white matter tracts altered in aging are also the most susceptible to schizophrenia [46]. These findings may collectively implicate premature aging of oligodendroglia in schizophrenia as they constitute over half of all cells present within the white matter.

Age-related loss of brain connectivity underlies cognitive decline, following a “last in, first out” pattern, whereby white matter tracts associated with cognition are the last to be fully myelinated and the first to be lost in aging [47,48,49]. This phenomenon, termed “white matter retrogenesis”, is characterised by heterochronicity and spatial heterogeneity across the tracts. Overall, this suggests that the latest tracts to develop are the most vulnerable to the detrimental effects of aging [50,51,52,53]. In particular, post-mortem diffusion Magnetic Resonance Imaging studies indicate ontogenetic differences between early-myelinating projection and posterior callosal fibres in aged individuals and schizophrenia patients [50,52]. A potential explanation for this phenomenon may lie in the fact that OLs are more susceptible to accumulating deleterious effects due to their comparatively elevated metabolic activity [25,54,55,56]. However, there is a lack of clear, unambiguous empirical data to confirm or refute these hypotheses. In this regard, diffusion tensor imaging studies in humans have demonstrated that aging initially affects white matter tracts associated with cognition, and this intrinsic susceptibility might play a crucial role in cognitive decline [41,44]. The susceptibility of cognitive decline and white matter alteration has been hypothesised to be due to age-related de-differentiation of brain connectivity [51,57]. This assumption posits that as aging occurs, cognitive white matter tracts lose their individuality, and shared microstructural alterations become more prevalent among all tracts. Furthermore, this apparent differential age trend supports the “last in, first out” hypothesis, where white matter tracts do not develop simultaneously. In fact, white matter tracts associated with cognition are the last to be fully myelinated and the first to degenerate [44,45,50].

Recent histological studies employing stereological approaches have confirmed approximately a 40% decrease in hippocampal OLs in schizophrenia patients compared to controls, across all ages. No significant changes in astrocyte or neuron numbers were observed [58], which may point to regional severities in the loss of OLs. Overall, further histological studies are needed to describe the changes in cellular quantification across different brain compartments. Nevertheless, recent transcriptomic studies at the single cell level have increasingly contributed to our understanding of aging processes in human and rodent OLs [23,24,59,60], whilst for schizophrenia, such studies are currently limited to neurons or astrocytes for the time being [61,62,63]. Moreover, due to the vast regional and cell-specific complexity of the human brain, a comprehensive transcriptional overview of the detrimental changes occurring during aging is still lacking. Thus, a major unfulfilled goal remains, i.e., obtaining a systematic view of the transcriptional and physiological changes across specific regions of the human brain to provide insights into the molecular pathways of aging.

### 1.3. Dissecting the Relationship with Aging- and Schizophrenia-Associated Genes Using Publicly Available scRNA-Seq Datasets

**Figure 1:**
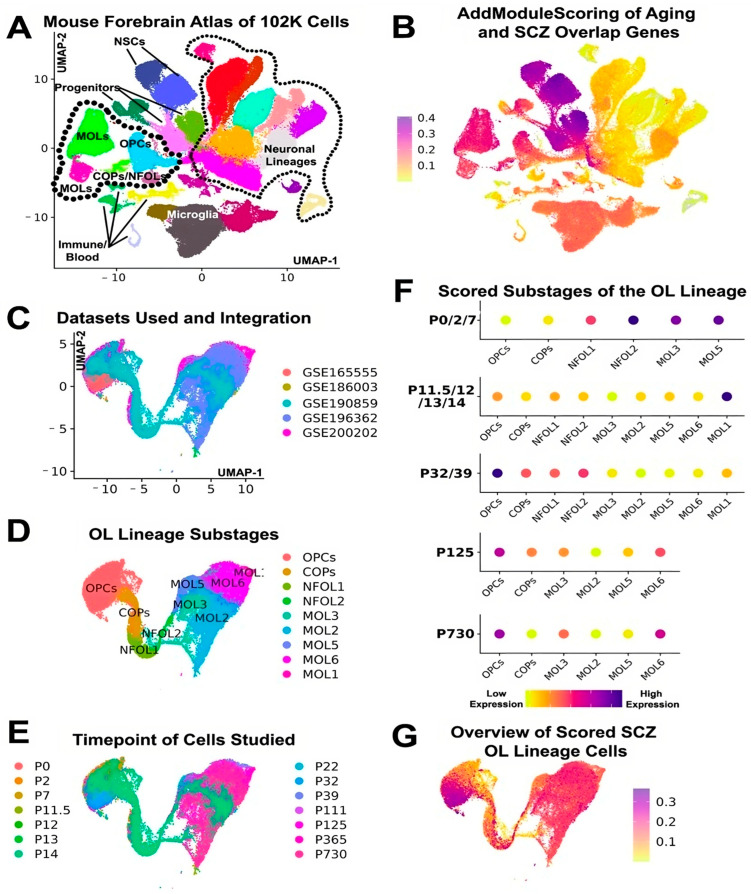
Exploring the aging- and schizophrenia-associated genes in OL lineage cells using publicly available scRNA-seq datasets. (**A**,**B**) UMAP plots illustrate the initial dataset of cells derived from the mouse forebrain comprising various neural lineages from E12.5 to 1 year of age. The AddModuleScore function through the processing of the 601 aging- and schizophrenia-associated genes as a geneset, reveals OL lineage cells as well as progenitors as the major population expressing those genes. (**C**–**E**) 5 different datasets as indicated with their GSE numbers in C were merged and integrated. OL lineage cells were extracted, which comprised of 30,000 cells at different stages of differentiation (**D**) and the developmental period (**E**). (**F**) AddModuleScore of the different stages of the OL lineage represented as dotplots for selected timepoints. Note: NFOLs were very rare in cells derived from mice older than 1 year of age. (**G**) Overview of the scores cells, which reveal that a subpopulation of OPCs from the second postnatal week have elevated gene expression levels of the aging- and schizophrenia-associated genes.

As a first step, to explore the plausible correlation of genomic factors that overlap between aging and schizophrenia (Figure 1A), relevant scRNA-seq datasets were re-analysed to identify the specific neural lineage, stage in differentiation, and developmental periods that show the extent to which schizophrenia risk genes are detected. Utilising an scRNA-seq atlas resource of over 100,000 cells across various lineages from the mouse forebrain, spanning from embryonic day 12.5 (E12.5) through to one year of life [64], we applied Seurat v5 for standard analysis procedures [65]. The ‘AddModuleScore’ function was used to calculate a ‘geneset’ score for each cell within the scRNA-seq dataset, based on the activities of schizophrenia risk genes. As evidenced by the literature and described above, OL lineage cells, as well as earlier progenitor/neural stem cell (NSC) stages, express surprisingly high levels of genes associated with aging and schizophrenia (Figure 1A,B). Upon verification of the increased expression of schizophrenia-associated genes in OL lineage cells, we further explored additional scRNA-seq datasets from different studies. These datasets were selected since they were generated from the same 10x v3 platform to investigate periventricular forebrain cells across a time span ranging from postnatal day 0 through to 2 years of age [64,66,67,68,69]. Initially, all datasets from various published studies were merged, and integration was carried out using the Single Cell Variational Inference (scVI) package in the python programming language in python [70]. This approach was aimed at procuring corrected count matrices and implementing Seurat’s ‘labeltransfer’ function. This function allows for inferring cluster labels from known cell types within the datasets (designated as the reference dataset) to another dataset (termed as the query dataset) based on mutual gene expression patterns. We utilised Seurat’s integration workflow to obtain Uniform Manifold Approximation and Projection (UMAP) plots and referred to the scVI and ‘sctransform’ method counts for subsequent analysis. For the purpose of this mini-review, we directed our attention to the OL lineage cells, representing approximately 30,000 cells that were very well integrated (Figure 1C–E).

Following the implication of the ‘AddModuleScore’ function, the data presented for individual OL stages across chosen ages in development unveiled dramatically distinct temporal changes and implied dynamic changes in the expression of schizophrenia risk genes not only from aging but also during development. These findings are in agreement with a study in which the expression of several OPC markers, including PDGFRA and NG2, was significantly downregulated in the prefrontal cortex of post-mortem schizophrenia brains [71] and corroborates our own recent findings with these markers as well as GPR17 being downregulated dramatically in mice [25].

Interestingly, another study by de Vrij et al. demonstrated that OPCs derived from induced pluripotent stem cells (iPSCs) of schizophrenia patients showed reduced differentiation capacity into mature OLs [72]. Our initial analysis, described in Figure 1, so far underlines that some of the molecular mechanisms leading to OPC dysfunction in schizophrenia could be attributed to the expression of common aging- and schizophrenia-associated genes.

## 2. Mechanistic Insights into OL Lineage Cells from Genome-Wide Omics: Further Bridging Aging and Schizophrenia

Through the initial findings described above in Figure 1, along with the emergence of high-throughput ‘omics’, it has become evident that mature OLs are not only marginally affected but that dysregulation along their lineage may further disrupt functionality in the central nervous system [63,73]. In comparison to neuroinflammatory conditions like multiple sclerosis, relatively little research has been conducted on OLs in the context of schizophrenia, as newer findings are emerging on the role of astrocytes and synaptic interactions [63]. We anticipate that combining and integrating multiple single-cell/nucleus RNA-sequencing studies to increase the cell numbers of OLs will undoubtedly yield insights into the transcriptional divergence during aging and schizophrenia. Nevertheless, much of our current knowledge has originated from earlier DNA-based, Single Nucleotide Polymorphism (SNP) genotyping and profiling of schizophrenia patient material [74,75,76]. SNP-associated genes range from 561 to 1650 enriched in OLs in the cited studies. Some of the observed differences in risk genes can be attributed to sample size and normalisation parameters. In the most extreme cases, applying more stringent criteria with various integrative tools revealed 41 ‘prioritised causal genes’, which may be used as novel biomarkers, including the downstream Wnt effector TCF7L2 [77], further discussed below.

A general conclusion from these high-throughput DNA/genome-wide expression studies (GWAS) is that identifying any schizophrenia-caused mechanism or potential upstream triggers leading to OL and myelin loss is highly complex and multifactorial, simply due to the large number of affected loci, which also seemingly leads to a concept of likely patient-specific phenotypes. With larger schizophrenia patient cohorts and healthy patients, the ideal future strategy using datasets from GWAS/transcriptomics/proteomics methods will involve subcategorising them based on clusters of segregated patients appearing on a 2D plot from dimensionality reduction parameters (i.e., tSNE/UMAP plots). This type of categorisation can uncover patient-specific mechanisms and help pave the way for personalised therapies. Newer techniques based on ‘predispositional’ whole genome sequencing, which aid in the identification of latent psychiatric conditions, may also serve as future early diagnostic tools, as has been proposed for other neurodegenerative diseases such as Alzheimer’s disease [78] (further discussed below in Section 5).

The general conclusions from the findings of genome-wide association expression studies, which are commonly accepted in the field, are that major myelin genes (MBP, PLP1, MOG, MAG, BCAS1, etc.) and the essential transcription factors (OLIG2 and SOX10) are all downregulated at the mRNA level in the prefrontal cortex of patients with schizophrenia [79,80,81], in addition to white matter (see below). These essential genes for the OL lineage are not only downregulated in psychiatric disorders but also in aging, as shown in numerous studies [23,24,59,60]. In the early pioneering studies performing bulk transcriptomics of brain tissue, the main outcomes were that aside from developmental-like gene ontology terms, other intriguing ones from the analysis include “cell communication”, “cellular process”, and “negative regulation of cell cycle” as the most differentially altered. Thus, multiple upstream mechanisms, together with genomic mutations, have an impact on OLs and myelin in schizophrenia patients. Furthermore, post-mortem studies from the prefrontal cortex of schizophrenia patients reveal that, although OPC densities remain unaltered in schizophrenia patients and age-matched individuals, their ability to differentiate compared to healthy individuals is severely hindered [82], resembling aged OPCs that fail to progress beyond the committed OL progenitors/COPs. The former authors performed microarrays of the matching studied tissue biopsies and found significant developmental pathways that are differentially altered (for example, TGFβ, Notch, Cell Cycle Regulation, Shh, Wnt, etc.) [82]. Since these experiments were performed on whole-laser microdissected tissues, it would be interesting for future follow-up studies to enrich for specific cell populations or to perform single-cell RNA sequencing to better resolve the transcriptomic alterations in aged individuals, which already display myelin deterioration in parallel with schizophrenia patients. These types of experiments can provide insights into the transcriptional correlation of OLs from the aged brain compared to their younger counterparts. Nevertheless, the aforementioned early transcriptomic data were highly informative on the developmental signalling mechanisms that appear to re-emerge later in the disease.

### 2.1. Insights from Upstream Receptor Signalling to Intracellular Processes in OLs in Schizophrenia Contexts

Several recent reviews have accentuated diverse pathomechanisms in OLs within the context of schizophrenia (for instance, see [83,84,85]). In this present review, we underscore evidence from more recent investigations, where we aim to describe shared aging and schizophrenia mechanisms. The sphingosine-1-phosphate (S1P) receptor, recognized as neuroprotective, experiences a global decline in the aging brain and plays a crucial role in promoting repair within the CNS (reviewed in [86,87]). A potential association has been recently proposed between upstream pathways, cell cycle arrest, and metabolism via the reduced protein levels of S1P, specifically in the white matter of schizophrenia patients [88]. Indeed, alterations in these lipid ratios could induce toxicity and death to OLs [88]. However, the exact downstream mechanisms are not fully elucidated, although a novel interaction may potentially involve redox-related metabolic pathways. In this context, redox-glutathione dysregulation during peak periods of developmental oligodendrogenesis has been observed to clinically manifest as schizophrenia symptoms in both human patients and in a schizophrenia mouse model [89]. The principal effector connecting redox alterations and schizophrenia is the well-defined Src kinase Fyn, which is crucial for myelin transport and assembly and is regulated by the redox-dependent transcription factor ERG1 [89]. The comprehensive scope of redox mechanisms in conjunction with decreased protein levels of S1P in schizophrenia uncovers compelling research opportunities. Given their significant impact on various biological processes and signalling pathways, these elements are promising and druggable targets for future investigations.

The ERBB/EGFR family of cell surface receptors represents another significant set of schizophrenia risk genes that act upstream to numerous biological processes and signalling pathways (comprehensively reviewed in [90]). The overactivation of ERBB/EGFR signalling at specific stages of the OL lineage precipitates programmed cell death/apoptosis and white matter loss, as indicated in previous studies ([83,91,92]). However, ERBB signalling knockout in mice also gives rise to schizophrenia phenotypes [93], which reinforces the view that the regulation of ERBB/EGFR signalling within OLs and myelin is complicated and complex. An additional aspect of ERBB/EGFR signalling involves Disrupted-in-Schizophrenia-1 (DISC1), one of the most studied schizophrenia risk genes. Interestingly, DISC1 is a direct target gene of ERBB2/3 specifically, excluding the other ERBB receptor isoforms [94]. Genetic targeting studies have indicated that DISC1 appears to act as an inhibitory factor for oligodendrogenesis [95]. These findings suggest that certain intracellular alterations result in aberrant activities at the protein level, culminating in detrimental effects. We anticipate that future newer high-throughput datasets at the atlas level will help resolve the precise triggers and provide further avenues for investigation.

### 2.2. Revisiting Publicly Available Datasets for Gathering Further Insights into Schizophrenia Pathology in OLs

**Figure 2:**
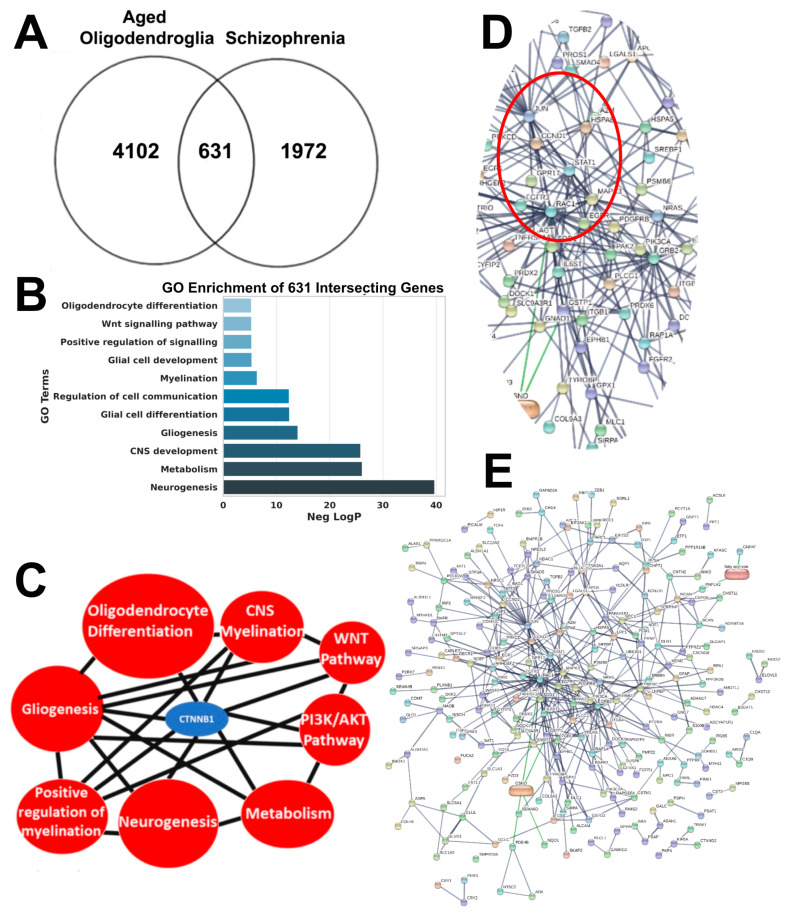
Transcriptomic correlation identifies common dysregulated networks associated with aged OLs and schizophrenia. The aging OL transcriptome was interrogated to identify novel associations with schizophrenia-specific databases (**A**). Neighbourhood-based entities set analysis (NEST) of OLs genes enriched in aging and associated with schizophrenia identified the downstream effector of Wnt signalling CTNNB1 as being associated in the alteration of crucial biological pathways such as gliogenesis, oligodendrocyte differentiation, and CNS myelination (PPI enrichment *p* < 0.0001) (**B**). Gene ontology (GO) analysis identified a number of biological processes commonly altered in aging OLs and schizophrenia (**C**). (**D**,**E**) Functional protein–protein network analysis (STRING V10.5) identified a central regulatory network including EGFR, JUN, GPR17, and RAC1. (**D**) is a zoomed cropped image of (**E**) at the central nodes to allow better visibility. Central nodes are selected by clustering nodes based on edge confidence score between 0 and 1, with a set minimum interaction score of 0.70. High confidence interaction between two nodes is represented by thicker edges. PPI enrichment *p* < 0.001.

Currently, various tools are available for the re-analysis of previously generated datasets from investigations published in the field, which can help unveil novel mechanisms previously missed as genomic databases expand over time. To this end, published datasets of genes altered in the aging brain and associated with OLs [95,96,97] were compared to a database of genes associated with schizophrenia (DISGNET, SZdatabase, and Psychiatry Genomic Consortium) [98,99]. This analysis identified 602 genes commonly altered in both groups (Figure 2A), which forms the basis for discussing the commonalities of aging and schizophrenia.

Among the identified genes were the late OPC gene GPR17 and major myelin genes, such as MBP, MOG, MOBP, and PLP (as described above), supporting the loss of white matter and myelin reported in imaging studies [100,101,102]. Next, we investigated the biological and molecular pathways that may be commonly co-altered in aging and schizophrenia. Gene ontology (GO) and pathway analysis identified terms such as neurogenesis, gliogenesis, glial cell development, and differentiation as commonly dysregulated in both aging and schizophrenia. This suggests that these common mechanisms are impaired and contribute to white matter loss in both disorders (Figure 2B,C) [25,56,103]. It is significant that changes related to neural cell differentiation are the top processes affected in glia, and the largest transcriptomic hub is related to the cell cycle, consistent with evidence that OPC self-renewal declines with aging [25,56].

This underpins the striking reduction in OPC numbers in the aging murine brain and age-associated loss of plastic responses of OPCs and GPR17+ cells to insults and damage [25,56]. Additionally, the analysis identified metabolism as a commonly altered biological pathway, further supported by evidence of elevated risk of diabetes and cardiovascular disorders in schizophrenia [22,104,105,106]. Furthermore, metabolic alteration is also suggested as a major contributing factor in the loss of OPC and myelin in aging [25,56,107]. GO analysis also highlighted several OL-specific pathways such as central nervous system (CNS) myelination, positive regulation of myelination, and oligodendrocyte differentiation. Perturbation of these pathways supports the hypothesis of impaired myelination and myelin loss reported in imaging studies of aging and schizophrenia (Figure 2B,C) [100,101].

Next, pathway analysis and Neighbourhood-based Entity Set analysis (NEST) were used to identify major molecular targets centrally connected to the functional pathways commonly altered in aging and schizophrenia [25,108]. Interestingly, NEST analysis highlighted CTNNB1, otherwise known as β-catenin (the transcriptional regulator form) (Figure 2B, Blue), shared between the pathways identified by GO analysis together with the WNT pathways and the PI3K/AKT pathway (Figure 2B). CTNNB1 is a master regulator of numerous biological processes, including cell proliferation and differentiation [109,110,111,112,113,114]. It is a downstream effector of the canonical WNT/GSK3β pathway. Interestingly, many mood stabilizers are GSK3β inhibitors [84], which are known to increase the transcriptional functions and stability of CTNNB1. In the case of schizophrenia, targeting GSK3β to indirectly enhance CTNNB1 activities as a transcriptional co-activator could be a possible therapeutic avenue for correcting any impaired gene expression critical to re/myelination, as described in our previous studies [109,111,115], given that GSK3β inhibitors are clinically used in other psychiatric and neurological conditions [116,117,118]. Moreover, further studies are required to exploit other druggable upstream highly connected intracellular regulators and their impact on transcriptional networks regulating OLs. To help fuel further laboratory investigations, the geneset representations common to aged OL and schizophrenia disease hallmarks were queried using the STRING db (V10.5) (Figure 2D), to resolve key functional protein–protein networks and reveal upstream regulators not known to be associated with schizophrenia pathology. These could emerge as potential therapeutic targets.

This analysis identified a core network with RAC1 as a central node interconnected with JUN, EGFR, MPK3, and STAT. Intriguingly, JUN and RAC1 are critical regulators of the actin cytoskeleton and are required for axonal ensheathment, synaptic modulation, and myelination [119,120,121,122], potentially revealing novel modes of dysregulation in OLs. RAC1 activation is essential for the nuclear translocation of CTNNB1 and subsequent gene activation due to strong gene regulatory effects of WNT signalling [123]. Furthermore, RAC1 overactivation resulting from Disrupted-in-Schizophrenia 1 (DISC1) downregulation is a major contributing factor in the loss of neuronal spines in schizophrenia patients [124,125]. Specific deletion of exon 3 of the DISC1-Δ3 gene in OPCs induced pathological OPCs in mouse models of schizophrenia, rather than myelin defects, which seemingly drive schizophrenia phenotypes. The loss of DISC1 functionality leads to a significant dysregulation of Wnt signalling in OPCs but can be rescued attenuation of Wnt ligands [86]. Additionally, inhibitors of the RAC1 downstream target p21-activated kinases (PAKs) can prevent synaptic loss and aberrant behavioral changes in animal models [124]. Notably, RAC1 acts as a regulator of OL differentiation and myelination [119,126], suggesting that RAC1 dysregulation may not only affect synapse maintenance but also contribute to white matter loss in schizophrenia.

Future studies should expand on these observations, using integrative multiomics to better clarify the impact of changes in OL lineage cells from patient material. Some of these future analyses, which would be most informative, should also be proteomic-based since these readouts may also provide additional biomarkers (discussed further below). Other future research we propose could employ spatially resolved transcriptomics to delineate the microenvironmental influences on OL lineage cells in schizophrenia, identifying niche-specific signals that contribute to disease phenotypes. Computational models analysing longitudinal multiomics data could predict the trajectory of OL lineage cell dysfunction, offering insights into the timing and progression of pathological changes.

## 3. Emerging Avenues in Schizophrenia Research

### 3.1. Dysregulated miRNAs Expression in OLs in Schizophrenia

miRNAs are small non-coding RNAs that play a crucial role in post-transcriptional gene regulation. Their dysregulation has been implicated in various neurological disorders, including schizophrenia [127]. In the context of OLs, miRNAs such as miR-219, miR-338, and miR-9 have been shown to be essential for OL differentiation and myelination [128]. Dysregulation of these and other miRNAs in schizophrenia might impact the expression of target genes involved in OL development and function, leading to the white matter abnormalities observed in schizophrenia patients [129]. A study by Lai et al. reported an altered expression of miRNAs in the prefrontal cortex of post-mortem schizophrenia brains, which were shown to target genes involved in myelination and OL function [130,131]. Similarly, miR-137, a critical risk factor for schizophrenia, is implicated in OL differentiation and myelin gene expression (reviewed in [132]).

### 3.2. Altered Neuroimmune Interactions Involving Microglia and Astrocytes in Schizophrenia

Neuroimmune interactions between microglia, astrocytes, and OLs are essential for maintaining CNS homeostasis and have been implicated in the pathophysiology of various neurological disorders, including schizophrenia [4,133,134]. Altered microglial activation and astrocyte function in schizophrenia can lead to disrupted OL development and myelination, contributing to the white matter abnormalities observed in patients [135,136]. A study by Bernstein et al. found that the complement component 4 (C4), a key player in synaptic pruning by microglia, is associated with an increased risk for schizophrenia [136]. Another study by Radulescu et al. reported that the gene expression of astrocytic markers, including GFAP, was altered in the prefrontal cortex of post-mortem schizophrenia brains [137], and are thought to be one of the defining pathological hallmarks of increased gliosis in schizophrenia patients [138]. Investigating the interactions between microglia, astrocytes, and OLs and how they might contribute to schizophrenia pathophysiology can provide new insights into disease mechanisms. Furthermore, given that these lines of investigations are intensely underway for other neurodegenerative diseases, there may be common therapeutic avenues for modulating pro-inflammatory responses.

### 3.3. Environmental Factors and Their Impact on OL Function in Schizophrenia

Environmental factors, such as prenatal infection, stress, and drug exposure, have been implicated in the etiology of schizophrenia and can also affect OL development and function (reviewed in [139,140]). Prenatal infection, for example, can result in the activation of the maternal immune system, leading to the production of pro-inflammatory cytokines that can affect fetal brain development, including OLs [141]. A study by Zhang et al. demonstrated that maternal immune activation in mice resulted in OL abnormalities and myelin deficits in the offspring [142]. Furthermore, prenatal stress has been shown to affect OL development and myelination in animal models [143]. Drug exposure, particularly the use of psychoactive substances such as cannabis, has been associated with an increased risk of developing schizophrenia [144] and can impact OLs. A study by Zamberletti et al. showed that adolescent exposure to delta-9-tetrahydrocannabinol (THC), the primary psychoactive compound in cannabis, led to long-lasting myelin deficits in the prefrontal cortex of adult rats [145]. Additionally, exposure to psychostimulants such as amphetamines can affect oligodendrogenesis and myelination [146,147]

## 4. The Growing Relationship of Schizophrenia with Other Neurodegenerative Diseases: Showcasing Multiple Sclerosis

Research increasingly shows a complex relationship between schizophrenia with multiple sclerosis, a chronic autoimmune disease with CNS inflammation and degeneration. Some studies, including that by Marrie et al., indicate an elevated risk of schizophrenia in multiple sclerosis patients, suggesting possible comorbidity [148]. Both conditions share clinical, epidemiological, genetic, and immunological commonalities, with inflammation and autoimmunity being central to their pathogenesis [149,150]. Benros et al. highlighted the bidirectional risk between schizophrenia and autoimmune diseases, including multiple sclerosis, pointing to shared immune response and inflammatory processes [151]. Alterations in white matter integrity and myelination are common to both, implying at least a degree of shared pathology [152], whilst studies underlying further mechanistic or genetic basis are still required to address this. The comprehensive review by Misiak et al. also outlines the challenges of using biomarkers from the two diseases despite the potential inflammatory degeneration of OLs [153].

The potential of identifying biomarkers shared between schizophrenia and multiple sclerosis opens avenues for early detection and intervention. The identification of 36 loci jointly associated with schizophrenia and multiple sclerosis, implicating genes involved in immune response and B-cell receptor signaling pathways, provides significant insight into their shared genetic underpinnings despite a negligible genetic correlation. This polygenic overlap highlights the importance of immune regulation processes in both diseases, revealing significant associations with immune-related genesets [152]. From such newer large-scale studies, the potential of applying biomarkers shared between schizophrenia and multiple sclerosis opens avenues for early detection and intervention [152,153], although probably further follow-up studies are required using multidisciplinary experimental approaches to confirm findings from genetic studies. There is no doubt that, as access to datasets from different sources, platforms, and technologies becomes easier, it will fuel integration possibilities to reveal common pathological hallmarks and biomarkers between diseases that were not previously apparent. A recent review from LoPresti also discusses the importance of serum biomarkers (namely neurofilaments and tau amyloid-peptide-β, Brl2, and Brl2-23, N-acetylaspartate, and 14-3-3 family proteins) as a testing ground for the early detection of multiple neurological conditions [154]. The intersecting pathological mechanisms of schizophrenia and multiple sclerosis are multifactorial, involving genetic, immunological, and pathophysiological pathways. While shared genetic markers and biomarkers offer promising insights for early detection, the multifactorial nature and diverse presentations of these conditions necessitate cautious interpretation and extensive research to validate these biomarkers’ roles in disease development and clinical applicability.

## 5. Looking to the Future for Schizophrenia Therapies and Summary

This review underlines the often-overlooked alterations in myelin within schizophrenia patients, highlighting a strong association with white matter atrophy, particularly in the context of aging phenotypes, where 602 genes that underpin this process were identified (Figure 2A). It underscores the significant disruptions experienced by OLs, the principal cell type involved in myelin production, in schizophrenia, with an emphasis on transcriptional changes. However, it acknowledges that genomic and epigenetic variations are equally critical, albeit less investigated. We discussed a spectrum of aberrant mechanisms in schizophrenia pathology, pointing to the variability in genetic and intracellular alterations across patients. Unravelling this potential heterogeneity could pave the way for identifying novel therapeutic targets by incorporating larger patient cohorts and advanced bioinformatic approaches. We proposed strategies to combine imaging with multiomics and pharmacogenomics [54,155,156,157] to drive patient-specific therapies that have already been used in other myelinopathies [158], Parkinson’s disease, [159] or determining gross structural changes in the brain [160]. The overall aim is to utilise this wealth of data for the development of therapeutic agents for schizophrenia together with machine learning approaches could streamline the process of identifying small molecules capable of rescuing impaired OLs in schizophrenia patients. Indeed, several small molecules identified to promote remyelination in mouse models could potentially be repurposed for use in schizophrenia patients (reviewed elsewhere [84]). In conclusion, a better understanding of the mechanistic and genetic basis for OL dysfunction in psychiatric diseases, combined with multidisciplinary approaches, holds promise for the discovery of therapies, addressing an unmet need in schizophrenia treatment.

## Data Availability

Data is available from the provided cited references and their GEO repositories.
